# Correspondence

**Published:** 1904-06

**Authors:** Albert Woldert

**Affiliations:** Rooms 3, 4 and 5, New Gary Buildings; Tyler, Texas


### Correspondence.

Rooms 3, 4 and 5, New Gary Buildings.
Tyler, Texas, May 24, 1904.

*Dr. F. E. Daniel, Editor Texas Medical Journal, Austin, Texas.*

Dear Doctor Daniel: In the *Journal American Medical Association* for May 21, 1904, page 1365, I observe that the Philadelphia County Medical Society at a recent meeting had under consideration the subject of having the American Medical Association to proceed against illegal practitioners throughout the country.

As secretary of different medical societies in this country for years, I have been brought into close relation with physicians here, and know their sentiments.

The first official act of our Smith County Medical Society was to examine the credentials of every doctor practicing medicine in the county. Am proud to say that I introduced that resolution. Our committee on legislation are still at work, and we find that a large proportion of the doctors in Smith county are either too lazy, or negligent or some other kindred feeling, to comply with the law. We hope to hand their names to the grand jury, after giving them a *large number of polite notices* to come up and register.

We occasionally have quacks to come amongst us, but, in order to proceed against them, we are first regulating ourselves. You are thoroughly familiar with the methods of quacks, and who at once pose as "martyrs" when any organized body of physicians proceed against them. However, if the American Medical Association would proceed against them, it would thus destroy their hidden power.

I know of no better way of building up the State Medical Association and the American Medical Association than to have these associations take an interest in local affairs. This would be a *mutuality* of interest in truth and in fact.

Won't you kindly write to Dr. Henry Bates (Fifteenth and Walnut Streets, Philadelphia, Pa., and get him to bring up this question before the American Medical Association? I have written Dr. Bates.

While attending the meeting of the State Medical Association at Austin recently, I had the pleasure of calling on the State Health Officer, Dr. Tabor, and in order to start the ball to rolling against getting rid of malarial fever in Texas, talked with him in regard to the objects of my paper which I read down there. I told him that malarial fever costs the people of Texas between $5,000,000 and $10,000,000 every year, and that 3074 people die with the disease annually. Further that yellow fever only killed ten or fifteen people every year, and that it seemed nearly all the money appropriated by the State was expended in getting rid of yellow fever. I also told him that I did not call on him for the purpose of asking him to relax his vigilance against yellow fever, but to try to induce him to begin some kind of movement towards getting rid of a disease in Texas which destroys over 3000 of us a year.

The other day I sent him some literature on this subject, and under date of May 16, 1904, he wrote me as follows:

"Dear Doctor: I thank you for yours of the 13th, enclosing papers relating to "Malaria in Texas." I am glad to note your interest in this matter and will be glad to receive literature at any time bearing on this subject. I hope to be able before long to distribute literature of this character among the profession and laity of this State. Yours very truly,

"George R. Tabor,
"State Health Officer."

I would like to have your help in this matter also. Very truly yours,

Albert Woldert, M. D.

